# Recent advances in the treatment of skin involvement in systemic sclerosis

**DOI:** 10.1186/s41232-017-0047-4

**Published:** 2017-06-12

**Authors:** Yoshihide Asano

**Affiliations:** Department of Dermatology, Graduate School of Medicine, University of Tokyo, 7-3-1 Hongo, Bunkyo-ku, Tokyo, 113-8655 Japan

**Keywords:** Interstitial Lung Disease, Dermal Fibroblast, Tocilizumab, Skin Fibrosis, Skin Sclerosis

## Abstract

Skin fibrosis is a devastating clinical condition commonly seen in skin-restricted and systemic disorders. The goal of skin fibrosis treatment is the restoration of abnormally activated dermal fibroblasts producing the excessive amount of extracellular matrix, which is generally a final consequence of the complex disease process including the activation of vascular and immune systems. Among various skin fibrotic conditions, the molecular mechanisms underlying dermal fibroblast activation have been mostly well studied in systemic sclerosis (SSc). SSc is a multisystem autoimmune and vascular disease resulting in extensive fibrosis of the skin and various internal organs. Since SSc pathogenesis is believed to include all the critical components regulating tissue fibrosis, the studies on anti-fibrotic drugs against SSc provide us much useful information regarding the strategy for the treatment of various skin fibrotic conditions. In the recent decade, as is the case with other autoimmune and inflammatory diseases, the molecular targeting therapy with monoclonal antibody has been clinically well examined in SSc. Promising clinical outcomes are so far reported in tocilizumab (an anti-IL-6 receptor antibody), rituximab (an anti-CD20 antibody), and fresolimumab (an anti-TGF-β antibody). The analysis of gene expression profiles in skin lesions of SSc patients treated with tocilizumab or fresolimumab revealed a critical role of monocyte-macrophage lineage cells in the development of skin fibrosis and the involvement of IL-6 and TGF-β in the activation of those cells. Considering that B cells modulate the differentiation and activation of macrophages, favorable clinical outcomes of rituximab treatment imply the central role of B cell/monocyte-macrophage lineage cell axis in the pathogenesis of SSc. This scenario may be applicable at least partly to other skin fibrotic conditions. In this review article, the currently available data on these drugs are summarized and the future directions are discussed.

## Background

Skin fibrosis is a devastating clinical condition resulting in severe disability and seriously affecting morbidity, which commonly occurs in skin-restricted and systemic disorders, including systemic sclerosis (SSc), localized scleroderma, and chronic graft-versus-host disease. It is widely accepted that constitutively activated dermal fibroblasts play a crucial role in the development and maintenance of skin fibrosis through the production of excessive amount of extracelluar matrix, but anti-fibrotic therapies targeting those cells generally elicit a limited effect on this pathological condition. In a sense, this is plausible because fibroblasts manifest a pro-fibrotic phenotype as a final consequence of the complex disease process consisting of complicated cell-cell interactions and networks of soluble factors. For instance, the fibrotic skin condition is generally related to T helper (Th)2/Th17-skewed immune polarization [1, 2], M2 macrophage differentiation [3], increased infiltration of plasmacytoid dendritic cells [4], increased endothelial intercellular adhesion molecule-1 expression [5], endothelial-to-mesenchymal transition [6], epithelial cell activation [7], and/or adipocyte-myofibroblast transdifferentiation [8]. In particular, autoimmunity and/or inflammation seem to play a central role because corticosteroids and/or immunosuppressants are effective for most of the skin fibrotic disorders even though clinical outcomes are variable in individual cases. Therefore, immune cells and several key molecules are the critical targets to interfere with the complex disease process underlying skin fibrosis. The molecular targeting therapy has recently caught much attention to achieve this goal and also would be helpful to further understand the pathogenesis of this clinical entity when favorable outcomes are obtained.

Among skin fibrotic conditions, the molecular mechanisms resulting in dermal fibroblast activation have been mostly well studied in SSc. SSc is characterized by extensive dermal fibrosis following aberrant activation of immune and vascular systems, in which all the critical components regulating tissue fibrosis are included [9, 10]. Therefore, the studies on anti-fibrotic drugs against SSc provide us much useful information regarding the strategy for the treatment of various skin fibrotic conditions. In the recent decade, as is the case with other autoimmune and inflammatory diseases, the molecular targeting therapy with monoclonal antibody has been clinically well examined in SSc. Promising clinical outcomes have been reported in tocilizumab (an anti-interleukin-6 (IL-6) receptor antibody), rituximab (an anti-CD20 antibody), and fresolimumab (an anti-transforming growth factor (TGF)-β antibody). In this review article, the currently available data on these drugs are summarized and the future directions are discussed.

### Tocilizumab

#### The role of IL-6 in SSc

Increasing evidence suggests a critical contribution of IL-6 to the development of tissue fibrosis and vasculopathy as well as inflammation associated with SSc. First, IL-6 is much more abundantly expressed in various types of cells, including dermal fibroblasts, dermal microvascular endothelial cells, inflammatory cells, and keratinocytes, of SSc lesional skin than in those cells of healthy control skin [11]. Consistently, the phosphorylation of signal transducer and activator of transcription 3 (STAT3), which is induced by the activation of IL-6 receptor/gp130 complex, is broadly detectable in various cell types, most remarkably in dermal microvascular endothelial cells, of SSc lesional skin irrespective of disease subtypes and disease duration, while totally absent or marginal in any cell types of healthy control skin [12]. More importantly, the elevation of serum IL-6 levels is associated with poor prognosis of this disease [11]. In in vitro studies SSc dermal fibroblasts seem to be activated by IL-6 in autocrine/paracrine manners [11, 13], and the activation of endothelial IL-6/STAT3 axis induces proliferation, migration, vascular instability, and endothelial-to-mesenchymal transition [14], all of which are characteristically seen in SSc endothelial cells [10]. With respect to the immunological aspect, IL-6 promotes the differentiation of Th2 cells and that of Th17 cells together with TGF-β [15], possibly contributing to the predominance of Th2 and Th17 cytokine production in SSc lesional skin [16]. These evidence strongly imply the possibility that tocilizumab modifies all the three cardinal pathological features of SSc, namely, inflammation, vasculopathy, and tissue fibrosis.

#### The effect of tocilizumab on SSc

Truly supporting the contribution of IL-6 signaling to SSc development, a favorable clinical effect of tocilizumab on skin sclerosis has been reported. Following two case series [17, 18], the detailed results of the faSScinate study (phase II trial of tocilizumab for SSc) was documented in 2016 [19]. After 24-week administration of tocilizumab (162 mg per each subcutaneous weekly injection), skin score estimated by Two-Gene SSc Skin Biomarker was significantly improved in diffuse cutaneous SSc (dcSSc) patients, who had disease duration of <5 years and IL-6-related inflammatory features (the elevation of C-reactive protein, erythrocyte sedimentation rate, or platelet count), compared with the placebo group. Based on this favorable clinical outcome, the global phase III trial is currently under the way with the larger number of SSc patients.

Another important finding in the faSScinate study was the alteration of gene expression profile in SSc lesional skin after the administration of tocilizumab [20]. The DNA microarray analysis with skin biopsy samples taken before and 24 weeks after the initial injection revealed that tocilizumab suppresses a cluster of genes related to M2 macrophages, suggesting a critical role of M2 macrophages in the development of skin fibrosis and a critical contribution of IL-6 to this process in SSc. M2 macrophages are derived from monocyte-macrophage lineage cells, which also provide a precursor of pro-angiogenic hematopoietic cells and fibrocytes [21, 22]. Indeed, in parallel with the reduction of skin sclerosis, the restoration of abnormal nailfold capillary changes and the healing of refractory digital ulcers were also reported after the administration of tocilizumab [12, 18]. Therefore, the target of tocilizumab treatment is at least partially the monocyte-macrophage lineage cells contributing to inflammatory, vascular, and fibrotic manifestations of SSc.

### Rituximab

#### The role of B cells in SSc

As represented by the SSc-specific sequential disease process, autoimmunity precedes the development of vasculopathy and tissue fibrosis, suggesting that aberrantly activated immune system plays a central role in the pathogenesis of SSc. At this moment, the direct role of SSc-related antinuclear antibodies, such as antibodies against topoisomerase I, centromere, and RNA polymerase III antigens, still remains unknown, but the close association of these antibodies with clinical manifestations suggests that altered B cell phenotypes possibly correlate with the central abnormality driving the progression of this disease through the genetic and epigenetic mechanisms shared with other cell types and/or the complex interaction with other immune and non-immune cells.

A critical role of aberrantly activated B cells has been implicated in the development of SSc-like features in murine animal models. Relevant to the elevated expression of CD19, a critical activator, in SSc B cells, *Cd19* transgenic mice exhibit hypergammaglobulinemia and autoantibody production due to the abnormal activation of B cells [23]. Tight-skin mice show hypodermal fibrosis, hypergammaglobulinemia, and positivity of anti-nuclear antibody and anti-topoisomerase I antibody, but both CD19 loss and B cell depletion by anti-CD20 antibody result in the reduction of these abnormalities [24, 25]. Supporting these findings, it is generally accepted that in addition to antibody production, B cells play multifaceted roles in immune system, such as cytokine production, antigen presentation, macrophage differentiation and activation, and lymphoid tissue development [26]. Consistently, B cell depletion therapy broadly affects disease processes of autoimmune diseases, such as rheumatoid arthritis, systemic lupus erythematosus, antinuetrophil cytoplasmic antibody-associated vasculitis, dermatomyositis/polymyositis, and primary Sjögren’s syndrome as well as SSc [27].

#### The effect of rituximab on SSc

In the first pilot study by Lafyatis et al. [28], 15 dcSSc patients with disease duration of <18 months were administered rituximab (1000 mg, twice, 2 weeks apart). In skin biopsy samples, the decrease in the number of myofibroblasts and skin-infiltrating B cells was evident at week 24 despite no significant change of modified Rodnan total skin thickness score (mRSS). In another pilot study reported by Smith et al. [29], 8 cases of dcSSc with disease duration of <4 years were administered rituximab (1000 mg, twice, 2 weeks apart) together with 100 mg methylprednisolone at each infusion. mRSS was significantly improved at week 24 compared with the baseline. Skin biopsy specimens taken at week 12 revealed the decrease in collage deposition and the number of myofibroblasts and skin-infiltrating B cells compared with those taken at the baseline. As a common finding in these two studies, no significant effect was detected on pulmonary function test results.

On the other hand, Daoussis et al. [30] performed a randomized controlled study of rituximab on 14 dcSSc patients, in which 8 patients were treated with two cycles of rituximab at baseline and week 24 (each cycle consisted of 4 weekly infusions (375 mg/m^2^)) and 6 patients received standard treatment alone. A year after the initiation of treatment, a significant reduction of mRSS was seen in the rituximab group, while not in the control group. More importantly, both of %FVC (forced vital capacity) and %DLco (diffusion capacity of the lungs for carbon monoxide) were significantly improved in the rituximab group, while no significant changes were seen in the control group. Similar favorable efficacy was reported in a nested case-control study using the European Scleroderma Trial and Research (EUSTAR) database [31]. In 63 SSc patients treated with rituximab, mRSS was significantly improved compared with closely matched control patient group. As well, %FVC was stabilized in the rituximab group, while not in the placebo group. Similar clinical effects of rituximab were recently reported by Daoussis et al. [32] in 51 SSc patients with interstitial lung disease (ILD). These three studies documented a potential disease-modifying effect of rituximab on skin fibrosis and ILD of SSc.

There is another report by Bosello et al. [33] regarding the long-term effect of rituximab in 20 SSc patients treated with rituximab (1000 mg, twice, 2 weeks apart). mRSS was significantly improved at 6 months and thereafter. As for ILD, among six patients with %FVC of <80%, %FVC was significantly improved from 64.3 to 71.0% at 1 year but decreased to 65.7% at the last follow-up period (mean follow-up of 48.5 +/− 20.4 months). The analysis of laboratory data displayed the recovery of B cells between 6 and 12 months, no change of serum IgG and IgA levels throughout the follow-up period, and a significant decrease in serum IgM levels at 6 months and thereafter. In some patients, relapse of skin sclerosis was attenuated by readministration of rituximab.

In addition, there are several case reports or case series in which calcinosis, digital ulcers, or arterial stiffness were improved by rituximab therapy [34–36]. Taken together, B cell depletion therapy is potentially able to modify the three cardinal pathological features of SSc, namely, fibrosis, vasculopathy, and autoimmunity. These results suggest that B cells are involved in the activation of vascular and fibrotic processes in addition to the activation of immune system in SSc.

### Fresolimumab

#### The role of TGF-β in SSc

TGF-β is a key growth factor regulating the activation status of dermal fibroblasts in SSc [37]. Although the expression pattern of TGF-β in the lesional skin of SSc is still controversial, TGF-β expression levels generally seem to be higher in patients with early and active disease, but weak or undetectable in patients with established skin fibrosis. So far, the expression profile of the three isoforms of TGF-β is generally understood as follows: (i) all the three isoforms of TGF-β are detectable in the extracellular matrix and (ii) the expression of TGF-β1 and TGF-β2 is most prominent around dermal vessels and is associated with perivascular infiltrating mononuclear cells [38–40]. Given that TGF-β action is determined by the state of activation and differentiation of the target cells and the presence and concentration of other cytokines and growth factors, TGF-β potentially promotes inflammation by recruiting leukocytes through the regulation of cell adhesion molecules and the creation of chemokine gradient, by activating leukocytes, and by inducing various pro-inflammatory cytokines and other mediators in early stage of SSc. In sclerotic stage, SSc dermal fibroblasts are constitutively activated with the pro-fibrotic phenotype quite similar to that of normal fibroblasts treated with TGF-β1 even though the expression of TGF-β is weak or undetectable in the skin [41]. This observation suggests that once activated, SSc fibroblasts establish a self activation system at least partially via autocrine TGF-β signaling. The increased expression of latent TGF-β receptors, including integrin αVβ3, αVβ5, and thrombospondin-1, contribute to this process in SSc dermal fibroblasts [42–46]. These receptors recruit and activate latent TGF-β on the cell surface and efficiently increase the concentration of active TGF-β around SSc fibroblasts. Therefore, dermal fibroblasts may be constitutively activated by autocrine TGF-β in SSc lesional skin. Thus, TGF-β is a promising therapeutic target of this disease.

#### The effect of anti-TGF-β antibody on SSc

A decade ago, the phase I and II clinical trials of metelimumab, a neutralizing antibody against TGF-β1, were conducted [47]. Forty-five dcSSc patients with disease duration of <18 months and moderate mRSS were treated with metelimumab (0.5, 5, or 10 mg/kg, intravenously, four infusions, 6 weeks apart) or placebo. Six months after the first infusion, no beneficial effect of metelimumab on skin sclerosis was observed. Taken it into account that all the three isoforms of TGF-β, especially TGF-β1 and TGF-β2, are highly expressed in the lesional skin of early and active SSc, the blockade of TGB-β1 alone seems to be insufficient to attenuate skin fibrosis of SSc. Indeed, all the three isoforms bind to the same receptors and exert similar biological effects on the proliferation, differentiation, and development of various cell types, and immune system. Therefore, the antibody blocking all the three isoforms was generated after this clinical trial.

In 2015, the result of phase II clinical trial of fresolimumab, a neutralizing antibody against TGF-β1, β2, and β3, was reported [48]. SSc patients with disease duration of <2 years and mRSS of equal or more than 15, who were on a stable dose of 10 mg/day or less of prednisone and no other immunosuppressants, were enrolled. Fifteen patients were treated with fresolimumab (twice [1 mg/kg], 4 weeks apart for 7 cases and once [5 mg/kg] for 8 cases), in which a case was withdrawn at week 4, and 4 cases were additionally treated with immunosuppressants during the safety follow-up period (one patient at week 9 and three patients at week 11). mRSS was significantly improved in both groups at week 11 and 17 compared with the baseline, while exacerbated at week 24. Consistently, in the analysis of gene expression profile in skin lesions, mRNA levels of the *THBS1* and *COMP* genes, which is included in 4 gene biomarkers [49], were decreased and reversed in parallel with the resolution and exacerbation of skin sclerosis, respectively. In addition, *CTGF*, *SERPINE1*, and *COL10A1* mRNA levels correlated with mRSS. Also, mRNA levels of the *CD14*, *CD163*, and *MS4A4A* genes, markers of monocyte-macrophage lineage cells, correlated with mRSS. Consistent with these results, the number of myofibroblasts was decreased after the treatment, though the thickness of the dermis was not changed.

As for the tolerability, bleeding and anemia were reported. Two cases experienced bleeding from gastric antral vascular ectasia which required blood transfusion. Bleeding from the gingiva, nose, and conjunctiva was also reported, and 10 out of 15 cases showed more than 10% decrease in hemoglobin levels during the study period. One patient died due to heart failure with severe cardiac fibrosis, though skin fibrosis was rapidly improved after receiving one dose of fresolimumab (5 mg).

This study firstly provided clear evidence that TGF-β is truly involved in the development of skin fibrosis in SSc and the blockade of all the three isoforms of TGF-β can be a therapeutic strategy for skin sclerosis. In parallel with the decreased extracellular matrix production, monocyte-macrophage lineage-related gene expression was reduced, suggesting that TGF-β is involved in the skin infiltration of monocyte-macrophage lineage cells which play a critical part in the development of skin fibrosis as well as vasculopathy [21, 22].

## Conclusions

Although the detailed molecular mechanism leading to extensive tissue fibrosis still remains largely unknown in SSc, the favorable clinical outcomes of tocilizumab, rituximab, and fresolimuab provide us useful information to speculate the role of the key molecules and cells in its developmental process. The inactivation of monocyte-macrophage lineage cells in parallel of skin fibrosis resolution, which is commonly seen in SSc patients treated with tocilizumab and fresolimumab, strongly suggest the crucial role of monocyte-macrophage lineage cells and the involvement of IL-6 and TGF-β in the activation of those cells during the fibrotic process of this disease. Monocyte-macrophage lineage cells provide precursors of pro-angiogenic hematopoietic cells, the altered phenotype of which is associated with the development of SSc vasculopathy [21]. The restoration of nailfold vascular abnormalities by tocilizumab treatment supports a broad spectrum of roles of monocyte-macrophage lineage cells in SSc pathogenesis [12]. Considering that B cells modulate the differentiation and activation of macrophages [26, 50], B cell depletion therapy possibly acts on the pathological process targeted by tocilizumab and fresolimumab, namely, monocyte-macrophage lineage cells. At this moment, this is still just a hypothesis based on clinical outcomes, but further studies on the B cell/monocyte-macrophage lineage cell axis would shed new light on the molecular mechanism of tissue fibrosis in SSc, as well as other skin fibrotic disorders.

## Abbreviations

DLco: Diffusion capacity of the lungs for carbon monoxide
FVC: Forced vital capacity
IL-6: Interleukin-6
ILD: Interstitial lung disease
mRSS: Modified Rodnan total skin thickness score
SSc: Systemic sclerosis
STAT3: Signal transducer and activator of transcription 3
TGF: Transforming growth factor
Th: T helper
